# Neurochemical Signalling Associated With Gill Oxygen Sensing and Ventilation: A Receptor Focused Mini-Review

**DOI:** 10.3389/fphys.2022.940020

**Published:** 2022-07-13

**Authors:** Maddison Reed, Michael G. Jonz

**Affiliations:** ^1^ Department of Biology, University of Ottawa, Ottawa, ON, Canada; ^2^ Brain and Mind Research Institute, University of Ottawa, ON, Ottawa, Canada

**Keywords:** neuroepithelial cell, chemoreception, serotonergic receptors, hypoxia, cholinergic receptors, zebrafish

## Abstract

Despite the large body of work describing vertebrate ventilatory responses to hypoxia, remarkably little is known about the receptors and afferent pathways mediating these responses in fishes. In this review, we aim to summarize all receptor types to date implicated in the neurotransmission or neuromodulation associated with O_2_ sensing in the gills of fish. This includes serotonergic, cholinergic, purinergic, and dopaminergic receptor subtypes. Recent transcriptomic analysis of the gills of zebrafish using single-cell RNA sequencing has begun to elucidate specific receptor targets in the gill; however, the absence of receptor characterization at the cellular level in the gill remains a major limitation in understanding the neurochemical control of hypoxia signalling.

## Introduction

Adequate oxygen delivery to tissue is important for maintaining cellular processes and homeostasis. Thus, an animal’s ability to detect low oxygen, and respond appropriately, is crucial for survival. Vertebrate respiratory chemoreceptors detect such chemical changes in the environment, or arterial blood supply, and upon stimulation initiate corrective autonomic reflexes, such as the hyperventilatory response (HVR) to hypoxia (Perry et al., 2009; Nurse, 2010; Jonz, 2018). The most well described vertebrate chemoreceptors are the type 1 (or glomus) cells of the mammalian carotid body. Hypoxic activation of these cells involves the modulation of K^+^ conductance, membrane depolarization, and resulting Ca^2+^-dependent neurotransmitter release to act on sensory terminals of the carotid sinus nerve (Lopez-Barneo et al., 1988; Gonzalez et al., 1994; Nurse, 2010; Kumar and Prabhakar, 2012).

The neurochemistry involved in transmitting the hypoxic signal in the carotid body is well characterized. Adenosine triphosphate (ATP) is released by type 1 cells to act on afferent terminal purinergic P2X3 receptors, and is one of the main excitatory neurotransmitters involved in hypoxic signalling (Zhang et al., 2000; Nurse, 2005). Adenosine, produced by the breakdown of extracellular ATP, acts on adenosine A2a receptors to further enhance the hypoxic response by type 1 cells (Nurse, 2014). Acetylcholine (ACh), co-released with ATP, is also largely responsible for excitatory signalling in the carotid body *via* post-synaptic nicotinic receptors (Zhang et al., 2000). Dopamine, acting on D_2_ receptors, plays a modulatory role in mediating the response to hypoxia in the carotid body (Benot and López-Barneo, 1990; Iturriaga and Alcayaga, 2004). Additionally, embedded within the respiratory epithelium of neonatal mammals lie clusters of chemosensory neuroepithelial bodies (NEBs), of which serotonin (5-hydroxytryptamine, 5-HT) is the major neurotransmitter released during hypoxia (Fu et al., 2002).

In contrast, remarkably little is known about the receptors and afferent pathways mediating HVR in fishes. In this review, we aim to summarize the available evidence that has linked specific receptor types in the gills to neurochemical signalling associated with O_2_ sensing in fish.

## Fish Oxygen Chemoreceptors

Neuroepithelial cells (NECs) were first identified in the gill by Dunel-Erb et al. (1982), who noted a similar morphology to mammalian pulmonary chemoreceptors. NECs were characterized by the presence of dense-cored vesicles and contained the monoamine, serotonin (5-hydroxytrymptamine, 5-HT; Dunel Erb et al., 1982). Due to the homology between the carotid body and the first gill arch in fish, NECs are believed to be homologues of type 1 cells (Milsom and Burleson, 2007). More recent evidence has suggested they may have a closer evolutionary link to pulmonary NEBs (Hockman et al., 2017).

The first direct evidence for the involvement of gill NECs in O_2_ chemoreception came from studies using zebrafish (*Danio rerio*; Jonz et al., 2004). Using whole-cell patch-clamp recordings of NECs isolated from the gills, it was shown that NECs responded to a decreased PO_2_ by inhibition of background K^+^ channels and membrane depolarization. Subsequent studies showed similar O_2_ sensitivity of gill NECs in channel catfish (*Ictalurus punctatus*; Burleson et al., 2006), and isolated NECs from adult goldfish (*Carassius auratus*) responded to hypoxia by Ca^2+^-dependent vesicular recycling (Zachar et al., 2017), which is consistent with neurotransmitter release and activation of sensory nerve fibres to initiate the hypoxic response. Currently there is no direct evidence of the neurotransmitters released during hypoxia and the receptors they act on remain uncharacterized.

## Receptor Control of Oxygen Sensing and Ventilation

Despite the lack of receptor characterization in the gill, there are numerous studies identifying neurotransmitters and neuroendocrine factors that may be associated with O_2_ sensing in the gills of fish (recently reviewed by Porteus et al., 2012; Pan and Perry, 2020). The following sections will summarize the major candidate receptor subtypes suggested to play a role in facilitating and mediating the ventilatory responses to hypoxia. These studies are summarized in Table 1.

**TABLE 1:** Summary of receptor types reported to effect changes in ventilation amplitude and/or frequency and their location within the gill, if available. The first column indicates receptor type, as characterized by pharmacological studies, gene expression analysis or immunohistochemistry.

Receptor type	Effect on ventilation^a^	Location within gill	References
**Serotonergic receptors (5-HT)**			
α-methyl-5-HT (5-HT_2_ receptor agonist)	↑		McDonald et al. (2010)
Ketanserin (5-HT_2_-R antagonist)	↓		McDonald et al. (2010), Shakarchi et al. (2013)
1-phenylbiguanide (5-HT_3_ receptor agonist)	↑		Janvier et al. (1996)
Metoclopramide (5-HT_3_ receptor antagonist)	↓		Janvier et al. (1996)
MDL72222 (5-HT_3_ receptor antagonist)	↓		Jonz et al. (2015)
*CR759836.1* (gene encoding 5HT_3A_-like receptors)		Neurons	Pan et al. (2022)
*htr1ab* (gene encoding 5-HT_1A_)		NECs	Pan et al. (2022)
**Cholinergic receptors (ACh)**			
Nicotine (nicotinic ACh receptor agonist)	↑		Burleson and Milsom (1995a)
Hexamethonium (nicotinic ACh receptor antagonist)	↓		Shakarchi et al. (2013)
Muscarine (muscarinic ACh receptor agonist)	↑		Burleson and Milsom (1995a)
Atropine (muscarinic ACh receptor antagonist)	No effect		Burleson and Milsom (1995a), McKenzie et al. (1995), Burleson and Smatresk (1990)
	↓		Rahbar et al. (2016)
*chrna7* (gene encoding nicotinic ACh receptor subunit α7)	-	NECs	Lauriano et al. (2021)
*chrna2b* (gene encoding nicotinic ACh receptor subunit α2b)	-	NECs/neurons	Pan et al. (2022)
*chrna6* (gene encoding nicotinic ACh receptor subunit α6)	-	NECs	Pan et al. (2022)
*chrnb3a* (gene encoding nicotinic ACh receptor subunit β3a)	-	NECs	Pan et al. (2022)
*chrnb4* (gene encoding nicotinic ACh receptor subunit β4)	-	Neurons	Pan et al. (2022)
**Purinergic receptors**			
ATPγS (broad spectrum purinergic receptor agonist)	↑	-	Coe et al. (2017)
PPADS (purinergic P2X2/3 receptor antagonist)	↓	-	Coe et al. (2017)
P2X3 immunolabelling	-	NECs/neurons	Jonz and Nurse (2003), Rahbar et al. (2016)
Aminophylline (adenosine A1/2 receptor antagonist)	↓	-	Stecyk and Farrel (2006), Stensløkken et al. (2004)
SCH58261 (adenosine A2a receptor antagonist)	↓	-	Coe et al. (2017)
**Dopamine receptors**			
DA	↓	-	Shackarchi et al. (2013)
*drd4a* (gene encoding dopamine D_2_-like family D_4_ receptor)	-	NECs	Pan et al. (2022)
*drd3* (gene encoding dopamine D_2_-like family D_3_ receptor)	-	Neurons	Pan et al. (2022)

a Effect on ventilation measured as amplitude or frequency.

### Serotonergic Receptors

Serotonergic receptors are divided into seven distinct classes, 5-HT_1-7_, largely based on their structural and operational characteristics within the serotonergic system (Peroutka and Howell, 1994; Hoyer et al., 2002). 5-HT receptors belong to the G-protein-coupled receptor (GPCR) superfamily, with the exception of the 5-HT_3_ receptor, which is a ligand-gated ion channel (Hoyer et al., 2002). Of the seven distinct classes, there are also a number of different 5-HT_1-7_ receptor subtypes, each having a distinct pattern of distribution and function in the nervous system, giving rise to many different signalling capabilities (reviewed by Barnes and Sharp, 1999). Some post-synaptic 5-HT receptor subtypes are known to cause neuronal depolarization (5-HT_2A_, 5-HT_2C_, 5-HT_3_ and 5-HT_4_ receptors) leading to excitatory signalling, or neuronal hyperpolarization (5-HT_1A_ receptor) inhibiting signalling (Barnes and Sharp, 1999).

As NECs characteristically contain 5-HT, it has been long hypothesized that 5-HT is involved in the neurotransmission of the hypoxic signal. Early evidence of this came from isolated gills of rainbow trout (*Oncorhynchus mykiss*), where 5-HT elicited a modest transient burst of chemoreceptor activity (Burleson and Milsom, 1995a). Additionally, rainbow trout intra-arterial injections of 5-HT also caused an elevation in gill ventilation and heart rate (Burleson and Milsom, 1995b).

Pharmacological studies targeting specific 5-HT receptor subtypes have revealed a potential excitatory role of 5-HT_2_ and 5-HT_3_ receptors. In the European eel (*Anguilla anguilla*), intravenous administration of 5-HT elicited a large increase in ventilatory frequency and amplitude (Janvier et al., 1996). Janvier et al. also showed the 5-HT-induced hyperventilatory response could be mimicked by the 5-HT_3_ receptor agonist, 1-phenylbiguanide, and blocked by the 5-HT_3_ receptor antagonist, metoclopramide. In the Gulf toadfish (*Opsanus beta*), injection with the 5-HT_2_ receptor agonist, α-methyl-5-HT, increased ventilation amplitude and this response was attenuated by the 5-HT_2_ receptor antagonist, ketanserin (McDonald et al., 2010). Similarly, 5-HT mimicked hypoxia by increasing ventilation frequency of 7 days post-fertilization (d.p.f) zebrafish larvae, and subsequent addition of ketanserin reversibly reduced ventilation frequency (Shakarchi et al., 2013). In zebrafish larvae, 5-HT_3_ receptor blockade with tropanyl 3,5-dichlorobenzoate (MDL 72222) also reduced the hyperventilatory response to acute hypoxia (Jonz et al., 2015). These results suggest an excitatory role for 5-HT_2_ and 5-HT_3_ receptors; however, it is unknown if these effects were limited to the gills, as whole animal experiments do not exclude potential targets in the central nervous system (CNS). Additionally, in the gills of the Antarctic fish (*Pagothenia borchgrevinki*), branchial vasoconstriction was mediated by 5-HT_2_ receptors (Sundin et al., 1998); and previous studies demonstrated the innervation of the filament arteries by serotonergic neurons in trout and zebrafish (Bailly et al., 1989; Jonz and Nurse, 2003). Given the effects of hypoxia on blood flow in the gills (Booth, 1979), an additional role of 5-HT_2_ receptors in controlling vascular responses to hypoxia is conceivable.

Recent single-cell transcriptomic analysis of chemosensory NECs and other cell types of the gills of adult zebrafish has begun to provide compelling evidence for potential actions of these receptor types by localizing them to, and within, the gill. The gene encoding 5-HT_3A_-like receptors shows highest expression in gill neurons (Pan et al., 2022). As NECs are innervated by at least one population of intrabranchial neurons, as well as extrabranchial nerve fibres (Jonz et al., 2004), there is a plausible mechanism for chemotransduction *via* 5-HT_3A_ receptors in the gill. This would be similar to the carotid body, where post-synaptic excitation during hypoxia is mediated primarily by ionotropic 5-HT_3_ receptors and, to a lesser extent, 5-HT_2_ (Zhong et al., 1999; Nurse, 2005). Further work localizing 5-HT_3A_ receptors to the specific neuronal population innervating NECs would be needed to confirm this.

Interestingly, the gene encoding the inhibitory 5-HT_1A_ receptor, *htr1ab*, was found to be most abundant in NECs above any other cell type in the gill (Pan et al., 2022). This may suggest a specific O_2_-sensing role for these receptors, possibly through a negative feedback or autoreceptor mechanism, which has not yet been explored pharmacologically.

### Cholinergic Receptors

Cholinergic receptors are subdivided into nicotinic and muscarinic types. Nicotinic receptors (nAChRs) are ligand-gated ion channels formed by the assembly of five transmembrane subunits and mediate fast neurotransmission in the central and peripheral nervous systems (Ho et al., 2020). Many different nAChR subtypes exist, each consisting of a specific combination of subunits (α1-10, β1-4, γ, δ, and ε; Kalamida et al., 2007). Muscarinic receptors (mAChRs) are G-protein coupled receptors, of which five main subtypes exist (M1-5), and have actions in the central and peripheral nervous systems (Caulfield, 1993).

A role for ACh in gill neurotransmission is not as clear as in the mammalian carotid body. Non-serotonergic, NEC-like cells containing the vesicular ACh transporter, VAChT, have been identified in zebrafish (Shakarchi et al., 2013; Zachar et al., 2017) and mangrove rivulus (*Kryptolebias marmoratus*; Regan et al., 2011), though it is currently unclear if these cells function as O_2_ chemoreceptors. Ventilatory responses to ACh and nicotine have been reported in several species (Burleson and Milsom, 1995b; Shakarchi et al., 2013). The effects of muscarine, however, appear to be more uncertain. In rainbow trout, muscarine elicited moderate afferent nerve activity in gill arch preparations and increased ventilation frequency in whole animal experiments. The muscarinic antagonist, atropine, prevented stimulation by hypoxia; however, it had no effect on ventilation (Burleson and Milsom, 1995a; 1995b). Similarly, atropine had no effect on ventilatory responses to hypoxia in the Adriatic sturgeon (*Acipenser naccarii;* McKenzie et al., 1995) or the channel catfish (Burleson and Smatresk, 1990). In zebrafish, atropine abolished the ventilatory response to hypoxia, but only at very high concentrations (Rahbar et al., 2016). Administration of atropine also abolished the hypercarbia-induced ventilatory responses observed in Pacific spiny dogfish (*Squalus acanthias*; McKendry et al., 2001).

Additionally, there appears to be a time-dependent emergence of cholinergic control of ventilation during development. Exogenous application of ACh did not affect ventilation frequency in early stage zebrafish larvae (7–10 d.p.f); however, in late stage larvae (14–21 d.p.f) ACh had a stimulatory effect on ventilation frequency. The nicotinic ACh receptor antagonist, hexamethonium, did not inhibit hypoxia-induced hyperventilation at 10 d.p.f. but did after 12 d.p.f. (Shakarchi et al., 2013).

In the Asian catfish (*Heteropneustes fossilis*) the nicotinic receptor α7 subunit is expressed in the NECs and mucous cells in the gill and respiratory air sac (Lauriano et al., 2021). In addition, semi-quantitative PCR detected low expression of the AChR γ-like subunit in the gills of adult transparent *Pristella maxillaris* (Ma et al., 2021)*.* RNA sequencing of the adult zebrafish gills has further narrowed down potential ACh receptor subunit targets within the gill. The nicotinic receptor α2b subunit gene, *chrna2b*, was highly expressed in NECs and neurons; whereas the α6 subunit gene (*chrna6*) and β3a subunit gene (*chrnb3a*) were expressed primarily in NECs, and the β4 subunit gene (*chrnb4*) was mainly present in neurons (Pan et al., 2022). The location of these subunits supports a model in which VAChT-positive cells release ACh during hypoxic stimulation, leading to excitatory post-synaptic or paracrine effects on ACh receptors of neurons or NECs (Pan et al., 2022). Like NECs, VAChT-positive cells of the zebrafish gills are closely apposed to nerve fibres (Zachar et al., 2017).

### Purinergic Receptors

There are two families of purinergic receptors—P1 (adenosine) receptors, including four subtypes (A1, A2a, A2b and A3), and P2 (ATP) receptors, which are further subdivided into ionotropic P2X (P2X1-7) and metabotropic P2Y (P2Y1-12) receptors (reviewed by Burnstock, 2018). ATP is a major excitatory neurotransmitter in the mammalian carotid body (Zhang et al., 2000; Nurse, 2005), and adenosine, produced by the extracellular breakdown of ATP, enhances the excitatory response (Nurse, 2014). Such a role in the gills of fish has been less explored.

The broad-spectrum purinergic agonist, ATPγS, elicited a hyperventilatory response in zebrafish larvae. Further, the purinergic receptor antagonist, PPADS, which targets purinergic P2X2 and P2X3 receptors, inhibited the hyperventilatory response to hypoxia (Coe et al., 2017). Immunohistochemical staining of P2X3 receptors showed co-localization with NECs and 5-HT-positive neurons (Rahbar et al., 2016). P2X3 receptors were also found in 5-HT-positve cells in the tips of zebrafish lamellae (Jonz and Nurse, 2003).

In the common carp (*Cyprinus carpio*), purinergic blockade with aminophylline, an A1 and A2 receptor antagonist, reversed the increases in respiration rate that occurred with the onset of hypoxia (Stecyk and Farrell, 2006). Adenosine injection initiated a biphasic response in ventilation frequency (a decrease followed by an increase) in the epaulette shark (*Hemiscyllium ocellatum*), which was also blocked by aminophylline (Stensløkken et al., 2004). Moreover, in zebrafish, the A2a receptor antagonist, SCH58261, inhibited the ventilatory response to hypoxia (Coe et al., 2017).

The above studies provide evidence for an excitatory role of P2X2/3 and A1/2 receptors in control of the hyperventilatory response to hypoxia in fish. Further, these results suggest a similar mechanism to the carotid body, where pre- and post-synaptic A2a receptors are believed to enhance the response to hypoxia (Nurse, 2014).

### Dopaminergic Receptors

Dopamine receptors are G-protein coupled and include the D_1_-like receptor subtypes (D_1_ and D_5_) which activate adenylyl cyclase, and the D_2_-like subfamily (D_2_, D_3_, and D_4_) which inhibit adenylyl cyclase and activate K^+^ channels (reviewed by Martel and Gatti McArthur, 2020). Dopamine is an important inhibitory neuromodulator in carotid body hypoxia signalling (Nurse, 2010). Dopamine released by type 1 cells has an autocrine-paracrine action on dopaminergic D_2_ receptors located on type 1 cells to inhibit Ca^2+^ channels, leading to negative feedback regulation of further neurotransmitter release during hypoxia (Benot and López-Barneo, 1990).

Early evidence for a role of dopamine in the gills was shown in isolated gills of rainbow trout, where dopamine caused a small and brief burst in chemoreceptor activity followed by a mild inhibition of receptor discharge (Burleson and Milsom, 1995a). In zebrafish larvae, exogenous application of dopamine has been shown to decrease ventilation frequency as early as 7 d.p.f. (Shakarchi et al., 2013). RNA sequencing of the adult zebrafish gill detected dopamine receptors in the D_2_-like family, including *drd4a* in NECs and *drd3* highly expressed in neurons (Pan et al., 2022). This recent work using zebrafish suggests an inhibitory dopaminergic mechanism in the gill, possibly via D_2_-like receptors, and is an area for potential future developments. Pharmacologically targeting the specific dopamine receptor subtypes controlling the ventilatory responses to hypoxia may be an interesting extension of this work.

## Conclusion

Since the initial discovery of gill NECs, much work has been done to identify the neurotransmitters and their respective receptor types involved in mediating the physiological responses to hypoxia. Recent RNA sequencing has begun to further localize some of these receptor subtypes to NECs and neurons within the gill; however, future work is needed to continue to localize specific receptor subtypes within the gill and provide physiological evidence of their involvement in O_2_ sensing.
